# Genome-Wide Identification and Evolutionary Analysis of Receptor-like Kinase Family Genes Provides Insights into Anthracnose Resistance of *Dioscorea alata*

**DOI:** 10.3390/plants13091274

**Published:** 2024-05-05

**Authors:** Yuqian Jiang, Xin-Yu Lu, Ya-Li Qin, Yan-Mei Zhang, Zhu-Qing Shao

**Affiliations:** 1 School of Life Sciences, Nanjing University, Nanjing 210023, China; yuqian_j@outlook.com (Y.J.); qrm@smail.nju.edu.cn (Y.-L.Q.); 2 Institute of Botany, Jiangsu Province and Chinese Academy of Sciences, Nanjing 210014, China; luxinyu@jib.ac.cn; 3 Jiangsu Key Laboratory for the Research and Utilization of Plant Resources, Nanjing 210014, China

**Keywords:** *Dioscorea alata*, receptor-like kinases, *Colletotrichum gloeosporioides*, transcriptome

## Abstract

*Dioscorea alata*, commonly known as “greater yam”, is a vital crop in tropical and subtropical regions of the world, yet it faces significant threats from anthracnose disease, mainly caused by *Colletotrichum gloeosporioides*. However, exploring disease resistance genes in this species has been challenging due to the difficulty of genetic mapping resulting from the loss of the flowering trait in many varieties. The receptor-like kinase (RLK) gene family represents essential immune receptors in plants. In this study, genomic analysis revealed 467 RLK genes in *D. alata*. The identified RLKs were distributed unevenly across chromosomes, likely due to tandem duplication events. However, a considerable number of ancient whole-genome or segmental duplications dating back over 100 million years contributed to the diversity of RLK genes. Phylogenetic analysis unveiled at least 356 ancient RLK lineages in the common ancestor of Dioscoreaceae, which differentially inherited and expanded to form the current RLK profiles of *D. alata* and its relatives. The analysis of cis-regulatory elements indicated the involvement of RLK genes in diverse stress responses. Transcriptome analysis identified RLKs that were up-regulated in response to *C. gloeosporioides* infection, suggesting their potential role in resisting anthracnose disease. These findings provide novel insights into the evolution of RLK genes in *D. alata* and their potential contribution to disease resistance.

## 1. Introduction

*Dioscorea alata*, also called “greater yam”, “water yam”, “winged yam”, and “purple yam”, was initially cultivated in Southeast Asia and has since become a highly important crop in tropical and subtropical regions worldwide [1]. Among several cultivated yam species, *D. alata* stands out as one of the most extensively grown and produced yam species globally [2]. *D. alata* serves as a major source of food in tropical and subtropical regions, especially in West and Central Africa, where at least 60 million people depend on it [3]. It is also the second most important cultivated yam after *D. polystachya* in China [4]. *D. alata* is favored for its robust yield potential, thriving even in environments with low soil fertility. The popularity of *D. alata* stems from several advantageous traits, including ease of propagation, early vigor in outcompeting weeds, high starch, and protein content, excellent tuber storability, and a richness of anthocyanin in some varieties [3,5]. Furthermore, in addition to its food usage, *D. alata* is exploited for pharmaceutical products [6,7,8].

Despite these attractive advantages, the productivity of *D. alata* has always been influenced by many threads, including microbial pathogens and various pests [9,10]. One of the most important threads is the fungus *Colletotrichum gloeosporioides*, which causes anthracnose disease, resulting in leaf darkening, acute necrosis, premature shedding, and wilting [11,12], emerging as a primary concern affecting yield and post-harvest quality. It has been reported that *C. gloeosporioides* could even cause up to 90% yield losses [13]. Consequently, it engenders significant economic repercussions and necessitates frequent fungicide applications for management. However, the reliance on chemical control methods presents environmental hazards, the risk of fungicide resistance, and economic barriers for farmers in tropical regions [13]. Thus, there is an imperative to explore sustainable and effective strategies from a biological perspective to mitigate anthracnose and enhance the resilience of greater yam cultivation systems.

Breeding-resistant varieties of *D. alata* is an efficient way to control anthracnose disease. However, the genetic mapping-based cloning of *R* genes in *D. alata* is greatly impeded by the degeneration or loss of the flowering trait in many cultivars [14]. Therefore, although the genetic transformation of *D. alata* through different methods has been well established [15,16], the cloning of functional disease resistance genes (*R* genes) from this species with a forward genetic strategy is still challenging.

Proteins from the receptor-like kinase family compose one of the major types of plant cell surface immune receptors, which are involved in the recognition of pathogen-associated molecular patterns (PAMPs) from diverse types of pathogens [17,18]. The typical structure of RLK proteins is composed of a cytosolic kinase domain, a transmembrane domain, and diverse extracellular ectodomains (ECDs), such as leucine-rich repeats (LRRs), lectins and LysM motifs (LysMs). LRR-RLK is the biggest subfamily of RLK and contains several well-studied functional genes, including FLAGELLIN SENSING 2 (*FLS2*), EF-TU RECEPTOR (*EFR*) and *XA21* [19]. Taking advantage of the expansive knowledge of the significance of the RLK family in plant disease resistance, reverse genetic studies have been adopted to mine functional *R* genes against various pathogens in this family via genome-wide identification coupled with the expression analysis of RLK genes. Among them, several analyses have contributed to the identification of functional *R* genes. For example, a recent study identified an RLK family number, namely TaBIR1, contributing to wheat resistance against the fungus *Puccinia striiformis* f. sp. *tritici* [20]. Another study reported several RLKs as candidate *R* genes against anthracnose in common beans [21,22]. While these studies have highlighted the genome-wide study of RLK genes as an important reverse genetic strategy for mining *R* gene candidates, the genomic profile and expression responding to *C. gloeosporioides* of RLK genes in *D. alata* have not been explored yet.

In this study, the systematic analysis of RLKs in *D. alata* was conducted to uncover the family member composition, chromosomal distribution, evolutionary pattern, and dynamic expression during *C. gloeosporioides* infection. These results not only provide information that contributes to a deeper understanding of the RLK gene family evolution but also serve as valuable resources for further mining functional *R* genes and conferring anthracnose resistance.

## 2. Results

### 2.1. D. alata Genome Encodes a Large Number and Diverse Types of RLK Genes

**Figure 1:**
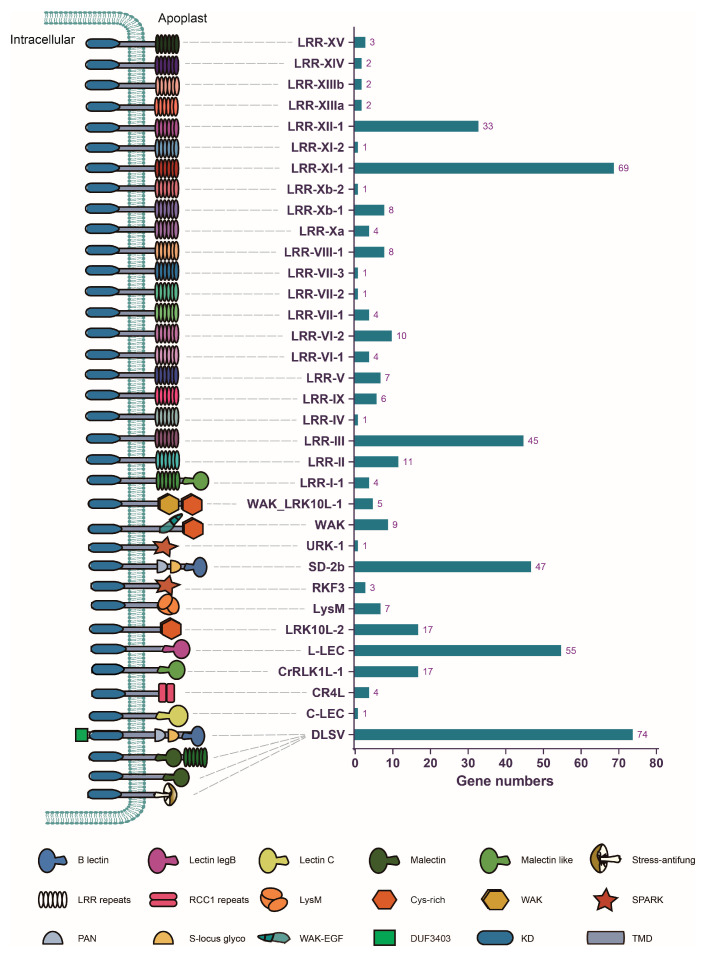
The number of RLKs in each subgroup. The schematic structure of each subgroup is shown on the left. The number of RLKs inside each subgroup are shown via the bar plot on the right.

A genome-wide survey of RLK genes was conducted in *D. alata* protein-coding genes, through which a total of 467 RLK genes were identified (Table S1). The identification of the RLK genes from three other species within the Dioscoreaceae family revealed that *D. alata* has the largest number of RLKs in this family (Table S2). Within all RLK protein sequences, different types of ECDs were identified, while LRR was the most prevalent one, presenting in 227 out of the 467 RLKs (Table S1). Previous studies have classified the plant RLK gene family into different groups according to their kinase phylogeny and ECD diversity [23]. To assign *D. alata* RLK genes into different subgroups, their protein sequences were subjected to blastp analysis against the reference RLK proteins from different subgroups [23]. The results showed that all *D. alata* RLKs are classified into 34 different subgroups (Figure 1, Table S1). Among them, the three largest subgroups are DLSV (74 genes), LRR-XI-1 (69 genes), and L-LEC (55 genes). Interestingly, DLSV, LRR-XI-1, and L-LEC also rank in the top three subgroups in other investigated species of Dioscoreaceae (Figure S1), indicating that extensive gene duplication occurred during the evolution of these subgroups. Most of the remaining subgroups only have a small number of genes, including 11 RLK subgroups that each possess no more than three genes. Notably, we found that four different types of the ECD structure existed in the largest subfamily, DLSV, which probably suggests that functional differentiation occurred in the DLSV subgroup.

All RLKs with LRR ECDs were classified into 22 subgroups, except for several of them in the DLSV subgroup (Figure 1 and Table S1). Phylogenetic analysis revealed that RLKs with the same ECD often form a monophyletic group, suggesting that they were duplicated from a common ancestor sequence. However, this is not the case for LRR-RLKs, which occupy several distantly related phylogenetic lineages and do not form a monophyletic group on the phylogeny (Figure S1), suggesting that they may arise from multiple domain fusion events.

### 2.2. Most D. alata RLKs Are Organized into Gene Clusters on Chromosomes

**Figure 2:**
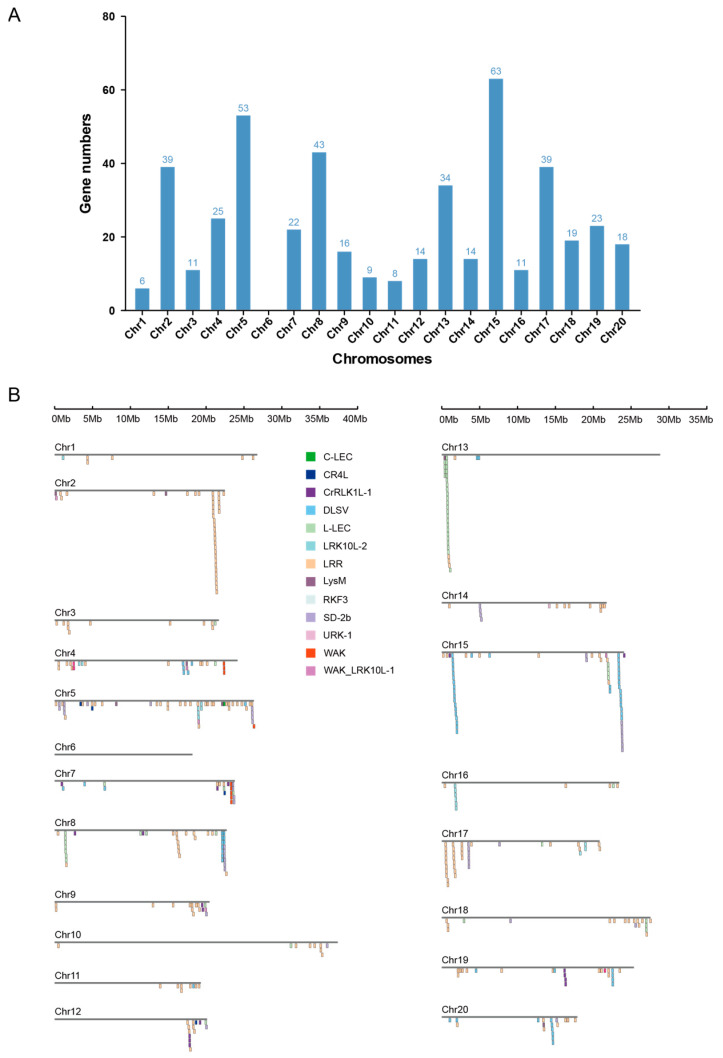
Physical distribution of RLKs. (**A**) The number of RLKs on each chromosome. (**B**) The physical map of RLK gene clusters on each chromosome. The RLK subgroups are shown in different colors.

The physical mapping of identified RLK genes was conducted to uncover their distribution on *D. alata* chromosomes. The results showed that the RLK genes were unevenly distributed across 19 of a total of 20 *D. alata* chromosomes (Figure 2A and Figure S3). Among them, chromosome 15 had the largest number of RLKs (63 genes), and chromosome 1 had the lowest number of RLKs (6 genes), whereas no RLKs were found on chromosome 6. Similar to previously reported distribution patterns of nucleotide-binding site-leucine-rich repeat (NLR, or NBS-LRR)-type *R* genes [24,25], RLKs were distributed unevenly not only between different chromosomes but also within a chromosome (Figure 2 and Figure S3). Of the 467 RLKs, 330 were presented on the chromosomes in 72 clusters, accounting for 70.7% of the *D. alata* RLKs. By contrast, only 137 genes presented as singletons. The biggest gene cluster was composed of 23 LRR-RLKs on chromosome 2. Other large clusters were also detected for the DLSV, LRR, L-LEC, SD-2b, and WAK subgroup genes (Figure 2B). The results suggest that tandem duplication may have contributed to the expansion of these RLK subgroups.

### 2.3. Ancient Whole Genome Duplications (WGDs) and/or Segmental Duplications (SDs) Contribute to RLK Diversification in D. alata

**Figure 3:**
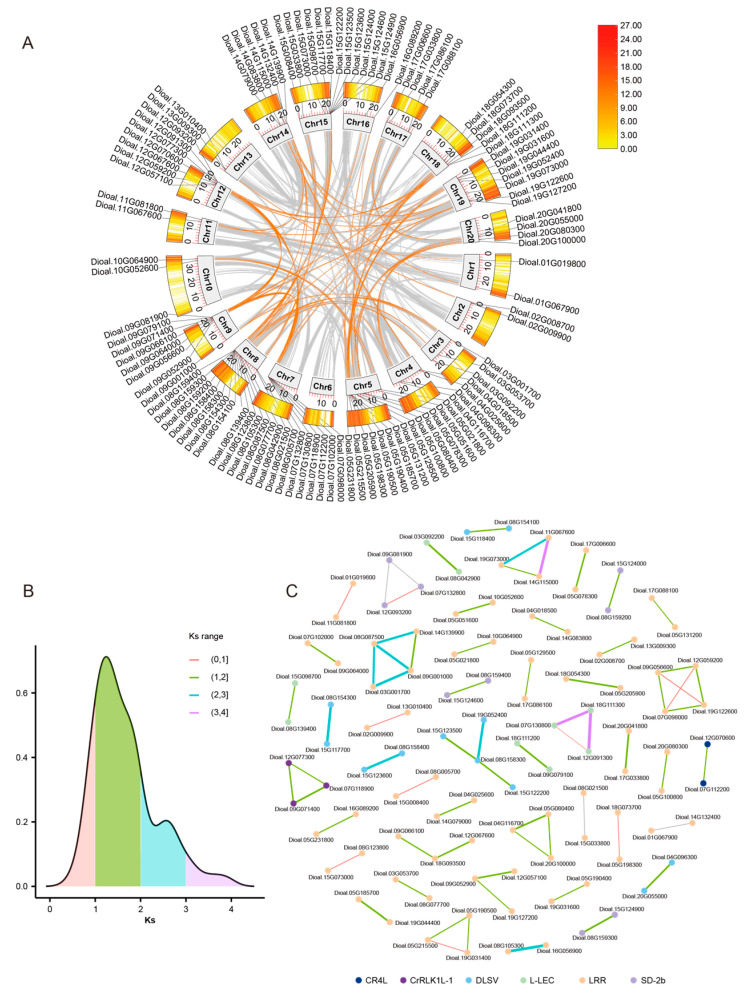
A considerable number of RLKs were duplicated through WGDs or SDs in *D. alata*. (**A**) The synteny analysis of RLKs among different chromosomes. The pairs of RLKs are shown by origin lines. (**B**) The distribution of Ks is shown by the density plot. Different Ks ranges are shown in different colors. (**C**) The plot of syntenic RLKs. The dot represents different RLK subgroups represented by different colors. The line shows the Ks value among each RLK pair. The color shows the Ks range, and the thickness shows the Ks value.

WGDs have been shown to consistently occur throughout plant evolution [26]. This factor, together with SDs, serves as another important force accelerating gene family expansion in addition to tandem duplication [27]. To explore the role of WGDs/SDs in *D. alata* RLK expansion, genome syntenic analysis was conducted. The results showed that 71 RLK pairs involving 106 unique genes may have duplicated through WGDs or SDs (Figure 3A), accounting for 23% of all RLKs in the *D. alata* genome. Among them, we detected several RLKs that displayed a syntenic relationship with more than one RLK gene, suggesting that multiple rounds of WGDs/SDs occurred to generate these gene pairs (Figure 3B). After classifying these genes into different RLK subgroups, the results showed that the 106 genes were from 6 different subgroups, including LRR (71 genes), DLSV (12 genes), L-LEC (9 genes), SD-2b (9 genes), CrRLK1L-1 (3 genes) and CR4L (2 genes); however, no WGDs/SDs were detected for RLKs from the remaining 7 subgroups.

The Ks value between a pair of genes is often used as a molecular clock parameter to estimate the divergence time [28,29]. To trace the age of these WGDs/SDs contributing to RLK expansion, we then calculated the Ks value of different RLK pairs and analyzed their distribution density (Figure 3B). The results showed that the Ks values of a large proportion of duplicated gene pairs were distributed in the range from 1 to 2 (Figure 3B), which indicates that duplication occurred 100 to 200 million years ago. In contrast, only a small number of gene pairs displayed values of Ks below one, suggesting that fewer recent WGDs/SDs contributing to RLK duplication occurred or were preserved during *D. alata* evolution.

Meanwhile, the Ka/Ks were calculated for each pair of genes, and none of them showed a value exceeding 0.5 (Table S3), which is the feature of strong negative selection. The results suggest that the functional differentiation of these different gene pairs might not have occurred after WGDs/SDs.

### 2.4. Phylogenetic Analysis and Evolutionary Trajectory of RLKs in D. alata and Its Relatives

**Figure 4:**
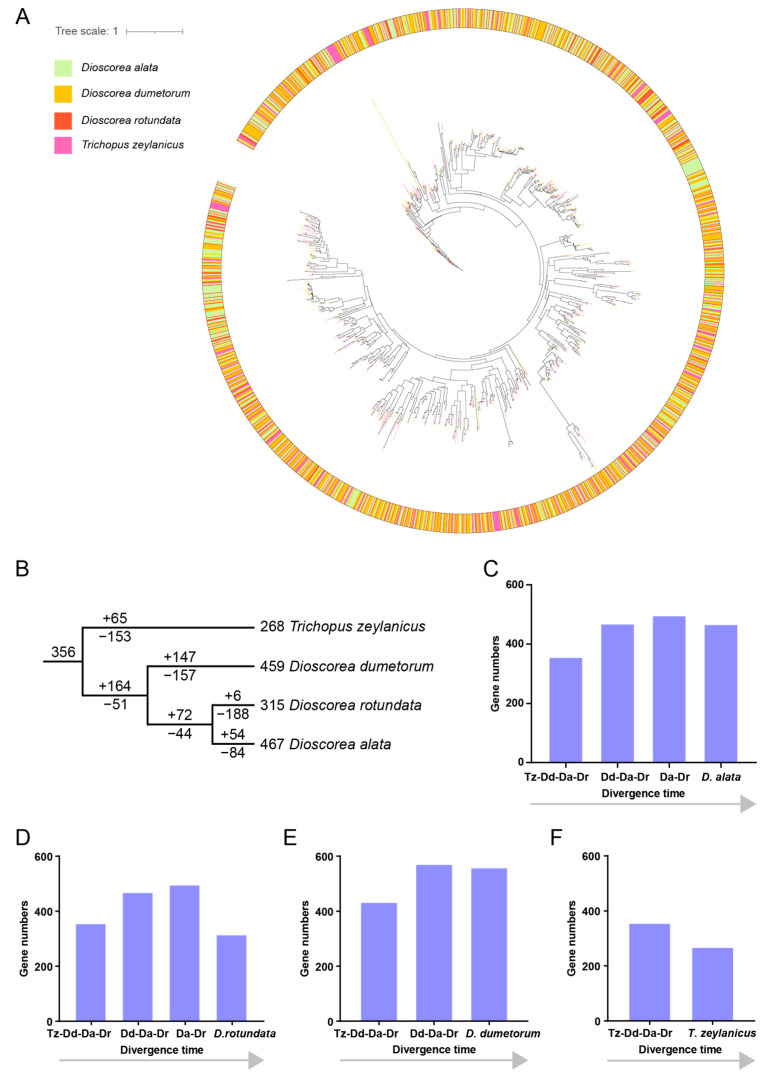
The phylogenetic analysis and evolutionary trajectory inference of RLKs from four species in Dioscoreaceae. (**A**) The phylogenetic tree of RLKs among four species from Dioscoreaceae. The outer ring is mapped by the color of each branch. (**B**) The gene duplications and losses inferred from the species tree and the gene tree. (**C**–**F**) The evolutionary trajectories of RLKs in the four species of Dioscoreaceae.

After noticing the contribution of anciently occurring WGDs/SDs in the RLK gene duplication of *D. alata*, we wondered how the RLK gene family evolved during the speciation of *D. alata* and its closely related species within the same genus and family. To reconstruct the evolutionary history of RLK genes during the evolution of Dioscoreaceae species, a phylogenetic analysis was conducted on RLK genes from four different species inside the Dioscoreaceae, which included *D. alata* (467 genes), *D. dumetorum* (459 genes), *D. rotundata* (315 genes) and *Trichopus zeylanicus* (268 genes). It was clear from the phylogeny that, despite the existence of some species-specific branches, a large number of RLK branches were presented in all four species; that is, they presented the common ancestor of Dioscoreaceae (Figure 4A). The results suggest that a large number of ancient RLK lineages diverged prior to the radiation of Dioscoreaceae. However, the absence of RKL genes could also be observed from one or more Dioscoreaceae species in many lineages (Figure 4A), suggesting that the species-specific loss of RLK genes also occurs frequently.

The evolutionary trajectory was then performed by the reconstruction of gene loss and gain events throughout the divergence of Dioscoreaceae species by reconciling the gene tree with the species tree. The results showed that RLK genes from the four Dioscoreaceae species could be traced as descendants of 356 ancestor genes in the most recent common ancestor of Dioscoreaceae (Figure 4B). The occurrence of different numbers of gene losses and gains at each node of Dioscoreaceae speciation contributed to the diverse profiles of RLK genes in the four species. For example, after the separation of the *T. zeylanicus* from the common ancestors of *D. dumetorum*, *D. alata*, and *D. rotundata* (defined as Dd-DaDr), 153 ancestral RLK genes were lost in the *T. zeylanicus* lineage (Figure 4B), whereas 65 genes duplicated from the remaining ancestral RLK genes were preserved in this lineage. However, only 51 ancestral RLK genes were lost in Dd-DaDr, whereas 164 new RLKs were duplicated from the remaining ancestral RLK genes (Figure 4B). Similar scenarios of loss and duplication of ancestral RLKs constantly happen at the lateral nodes of speciation. *D. dumetorum* lost 157 and duplicated 147 ancestral RLK genes after its separation with the common ancestor of *D. alata* and *D. rotundata* (defined as DaDr), whereas Da−Dr lost 44 and duplicated 72 ancestral RLKs after this divergence node (Figure 4B). Subsequently, after the separation of *D. alata* and *D. rotundata,* 84 and 188 gene losses and 54 and 6 gene duplications occurred in the two species, respectively (Figure 4B). It is clear that the contrasting extents of recent gene losses and duplications contribute to the great difference in the RLK gene number between *D. alata* and *D. rotundata*. 

Interestingly, after comparing the evolutionary trajectory of RLK genes in the four species from Dioscoreaceae (Figure 4C–F), we found a similar pattern of “first expansion and then contraction” in the RLK gene family evolution. These results not only suggest the ancient expansion of the diversity of the RLK gene family during the early period of Dioscoreaceae but also indicate the accelerated loss of ancestral RLK lineages and attenuate the duplication of new RLKs in the investigated Dioscoreaceae species.

### 2.5. The Profile of Cis-Regulatory Elements in the Promotor Regioin Suggests the Engagement of RLKs in Stimulus Responses

**Figure 5:**
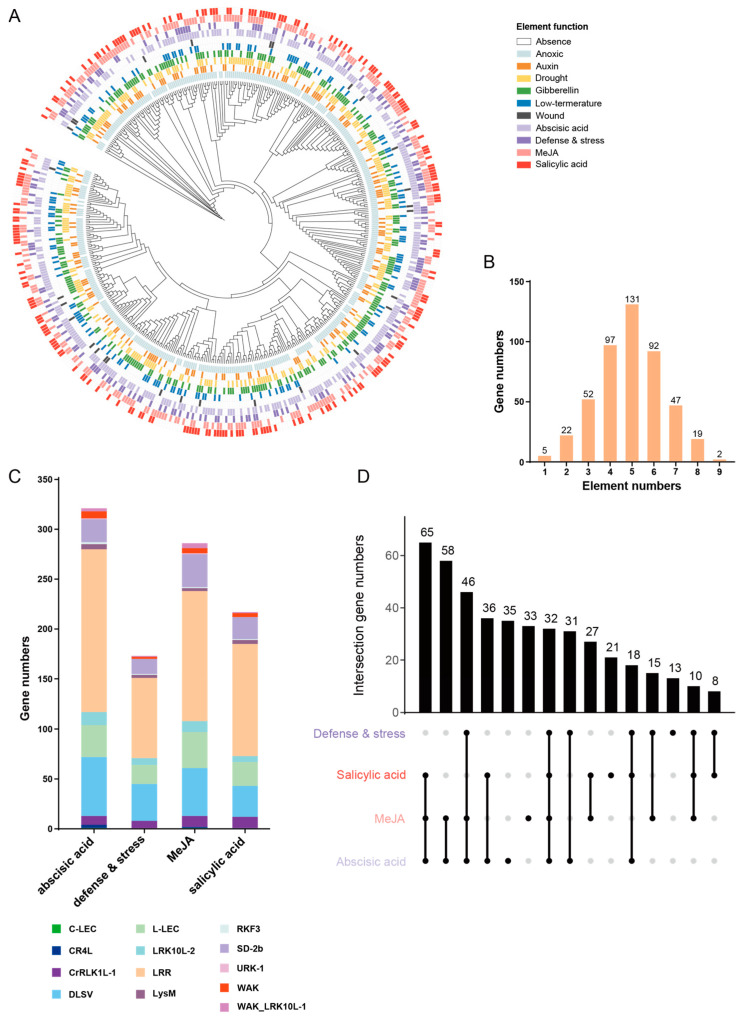
The cis-elements of different RLK genetic structures. (**A**) Cis-elements of different functions are shown in the outer ring. All RLKs are shown in the phylogenetic tree. (**B**) The number of RLKs with different cis-elements exist in their promoter region. (**C**) The number of RLKs in *D. alata* for each cis-element (RLKs may overlap in each group). (**D**) RLKs have four immune-related cis-elements, as shown by the up-set plot.

Previous studies have highlighted various functions of RLK family members, indicating that they serve as a major type of plant receptor in the recognition of biotic and abiotic stress stimulus signals from environments [19,30,31]. To gain insights into the function of *D. alata* RLKs in stress responses, we analyzed the presence of diverse types of stress-associated cis-regulatory elements, including those responding to auxin, defense and stress, gibberellin, low temperatures, salicylic acid (SA), abscisic acid (ABA), methyl jasmonate (MeJA), and droughts and wounds in the promoter region of *D. alata* RLK genes. The results show that the majority of RLK promoter regions had anoxic and abscisic-acid responsiveness elements, while only a very few RLKs had wound responsiveness elements (Figure 5A and Table S4). Meanwhile, almost all RLKs had more than two different cis-acting elements, except only five RLKs displayed only one element that responded to defense or salicylic acid (Figure 5B). The results suggest that the great majority of *D. alata* RLKs may be influenced and regulated by a variety of signaling pathways to precisely tune their expression.

To identify *D. alata* RLKs that are potentially involved in immune response, we paid more attention to genes possessing the following four types of cis-regulatory elements, including ABA, defense and stress, MeJA, and SA, respectively. We found that 321 RLKs responded to ABA, 286 RLKs responded to MeJA, 217 responded to SA, and 173 responded to defense and stress (Figure 5C and Table S5). We analyzed RLKs, which responded to four different stresses (Figure 5D). In RLK genes possessing the four cis-regulatory elements, the number of LRR-RLKs ranked first among all subgroups in each cis-regulatory element-containing RLK, followed by the DLSV subgroup. Notably, 32 RLKs possessed all four types of cis-elements, and 65 RLKs contained three different plant immune-related cis-regulatory elements, suggesting their high potential to participate in plant immune signaling.

### 2.6. Expression Analysis Identifies Candidate RLKs Involved in C. gloeosporioides Resistance

**Figure 6:**
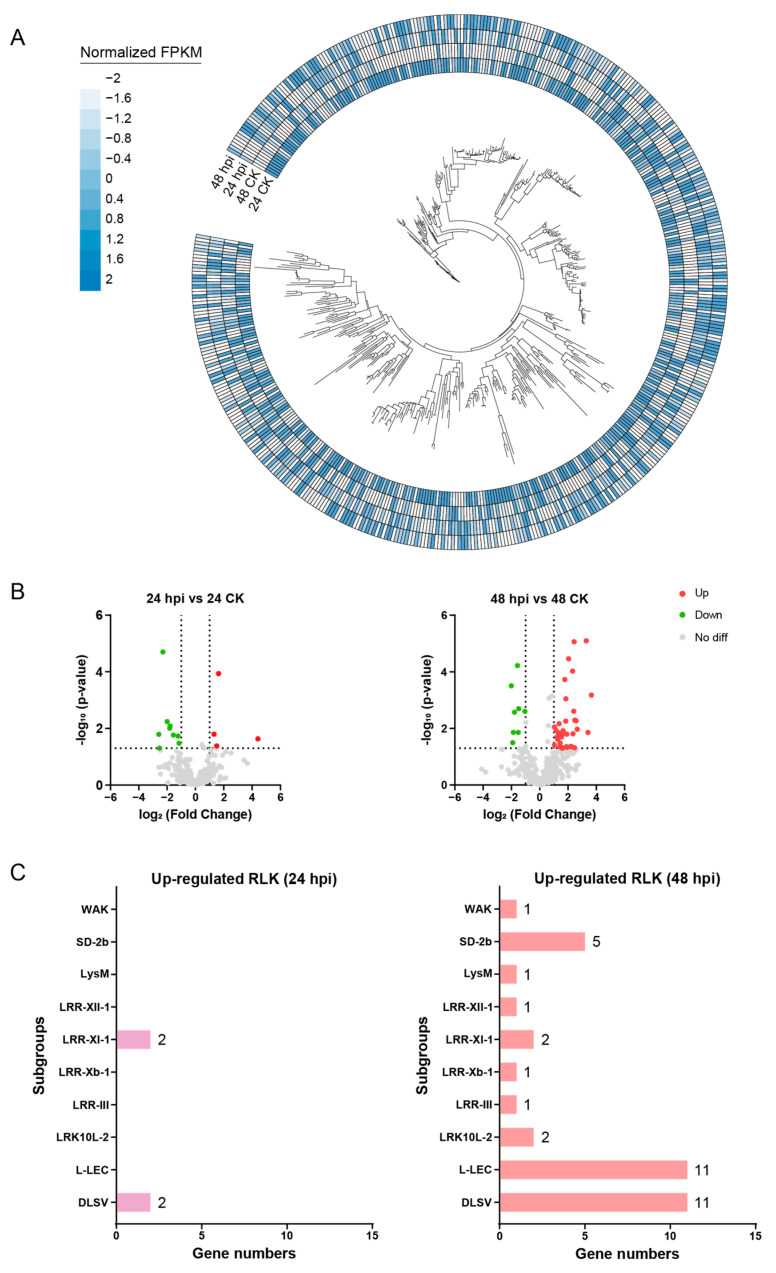
The expression analysis of RLKs responding to *C. gloeosporioides* infection. (**A**) Expression profile of each experimental group. Each RLK expression is shown by colors on the outer ring. The color represents the normalized FPKM. hpi, hours past inoculation. (**B**) The differentially expressed genes (DEGs) are shown by a volcano plot. (**C**) The number of up-regulated RLKs among each subgroup in 24 or 48 hpi.

Since RLKs have been widely reported as immune receptors of the first line in the plant immune system, we explored the potential involvement of *D. alata* RLKs in *C. gloeosporioides* defense by transcriptome analysis. After inoculating a resistant *D. alata* variety with *C. gloeosporioides*, the leaf samples were collected and subjected to transcriptome sequencing at 24 and 48 h post-inoculation (hpi), respectively, with leaf samples that were not inoculated by the pathogen as a control. While 451 of the 467 RLKs could be detected for their expression in either the pathogen-infected or the control samples (Figure 6A), a small number of RLKs displayed significant differential expression between the two groups of samples at either 24 or 48 hpi (Figure 6B). However, more differentially expressed genes (DEGs) emerged at 48 hpi than at 24 hpi (Figure 6B and Tables S6 and S7), suggesting the progressively enhanced immune response of *D. alata* against *C. gloeosporioides* infection.

Among the DEGs detected at the two time points, 4 and 36 displayed up-regulated expression at 24 and 48 hpi, respectively (Figure 6B), suggesting their potential participation in *C. gloeosporioides* resistance. Interestingly, the up-regulated genes showed no overlap between the two time points. It was shown that two RLKs from the LRR-XI-1 and two from the DLSV subgroup were up-regulated in 24 hpi treatment (Figure 6C). Although no RLKs from LRR-XI-1 showed up-regulated expression at 48 hpi, more up-regulated RLKs (11 genes) from the DLSV subgroup were detected at 48 hpi (Figure 6C). In addition, different numbers of up-regulated genes from the L-LEC (11 genes), WAK (1 gene), SD-2b (5 genes), LysM (1 gene), LRK10L-2 (2 genes), and LRR (5 genes) subgroups were detected at 48 hpi, indicating a much stronger immune response at 48 hpi.

## 3. Discussion

The RLK gene family represents one of the largest gene families within the plant genome and is also one of the major types of plant receptors responsible for the perception of diverse self and alien signals from abiotic and biotic stresses [19,30,31]. The comprehensive analysis of RLK genes in an increasing number of sequenced plant genomes has not only contributed to a better understanding of the origin, evolution, and functional divergence of this important gene family but has also provided invaluable resources for the identification of functional genes that are valuable for crop breeding [32,33,34,35,36,37]. In this study, the genome-wide identification and evolutionary analysis of RLK genes from *D. alata* provides additional insights into the evolution and functional roles of the RLK gene family, including its ancient divergence by means of considerable WGDs/SDs, rapid “birth to death” during speciation, and potential roles in disease resistance against anthracnose.

The first largest-scale evolutionary analysis of plant RLK genes can be traced to more than twenty years ago [38], which found several hundreds of RLK genes within a single plant genome. Subsequent studies revealed that the RLK gene family experienced rounds of ancient duplications to form dozens of ancient lineages [39]. The results from this study demonstrate that WGDs/SDs may have contributed significantly to the ancient expansion of RLK proteins, although the majority of RLKs may have emerged via tandem duplications, as displayed in gene clusters on *D. alata* chromosomes. In our study, over one hundred *D. alata* RLK genes were detected as the consequence of WGDs/SDs, with 78.1 percent of them displaying Ks values larger than one. The results suggest that many of these duplications did not occur specifically in *D. alata* but can be traced to more than 100 million years ago. The observed large number of WGDs/SDs (22.7 percent of all RLK genes in the genome) in the RLK gene family also indicates a fairly different expansion mechanism to that reported for the NLR immune receptor gene family [40]. While both of these gene families are dominated by tandem duplication in gene expansion, very few duplicated NLR genes were preserved after WGDs/SDs, especially at long-term evolutionary time. These results suggest that the two types of immune receptors may have evolved differently in terms of gene duplication mechanisms.

As a gene family responsible for responding to changing stress stimuli for the environments, it can be anticipated that dynamic gene loss and gain may have occurred during speciation. However, previous analysis of other gene families suggests that this process might be diverse and lineage-specific [41]. In this study, phylogenetic analysis of RLK genes across Dioscoreaceae species provides insights into the evolutionary trajectory of this gene family. Despite species-specific variations, a large number of RLK lineages appear to have originated from a common ancestor, predating the divergence of Dioscoreaceae. This is in accordance with previous studies on the RLK gene family in other plant lineages [39]. However, species-specific gene losses and duplications have contributed to the unique composition of RLK genes in each Dioscoreaceae species. The observed pattern of gene loss and gain suggests that dynamic evolutionary processes with the feature of “first expansion and then contraction” have shaped the RLK gene family during the speciation of *D. alata* and its Dioscoreaceae relatives.

Inspired by the increasingly successful applications of reverse genetic strategies in the identification of functional genes related to important traits of plants, many recent studies have performed the genome-wide analysis of the RLK gene family [21,42,43,44]. This serves as an important basis for following investigations to screen interested genes involving specific biological processes. For example, two RLK proteins have been identified as key regulators of arbuscular mycorrhizal symbiosis by comparative genomic and expression analysis in recent studies [32,33]. Several RLKs have been characterized to play positive roles against different pathogens infecting apples, *Nicotiana tabacum*, and wheat, taking advantage of the known RLK profiles in these species [34,35,36]. The genome-wide identification of RLK genes in *D. alata*, therefore, provides an important resource for this crop. Considering the fact that traditional genetic mapping is difficult in this species, this resource supports an alternative strategy for mining functional *R* genes of *D. alata* through the reverse genetic study. The presence of stress-responsive cis-regulatory elements in the promoter regions of RLK genes hints at their involvement in plant immune responses and several other types of stimuli. Interestingly, we found that 32 RLKs simultaneously possess four investigated immune-related elements, and 139 RLKs have three of them, suggesting their potential roles in plant defense. To identify potential genes that participate in *D. alata* resistance to the most severe disease, anthracnose, we conducted the transcriptome analysis of a *D. alata* resistant variety and identified 4 and 36 genes showing up-regulation after being challenged with *C. gloeosporioides* at 24 and 48 hpi, respectively. Of these, all up-regulated genes at 24 hpi and 34 of the 36 up-regulated genes at 48 hpi possessed at least one type of immune-related element at the promotor region, implying their roles in *C. gloeosporioides* resistance. In addition, previous studies have reported functional *R* genes of several LRR-RLK subgroups, including LRR-I, II, VIII-1, VIII-2, Xa, Xb, XI, XII, and XVI [31]. Interestingly, three LRR-RLK subgroups containing up-regulated RLKs in this study fell into these subgroups, further supporting their roles in *C. gloeosporioides* resistance. Taken together, although the further genetic transformation of a sensitive variety, *D. alata,* is required to validate their function, this study provides candidate genes involved in *C. gloeosporioides* resistance with multiple supporting examples of evidence.

In conclusion, our study provides a comprehensive overview of RLK genes in *D. alata*, offering new insights into their evolutionary history, genetic diversity, and potential roles in *C. gloeosporioides* resistance. These findings serve as the foundation resource for future research aimed at elucidating the molecular mechanisms underlying disease resistance and the stimulus perception of *D. alata,* facilitating the development of improved cultivars with enhanced resistance to *C. gloeosporioides* and other pathogens.

## 4. Materials and Methods

### 4.1. Genomes Used in This Study

Four species were used in this study, including *Dioscorea alata* [45], *Dioscorea dumetorum* [46], *Dioscorea rotundata* [47], and *Trichopus zeylanicus* [48]. The genome sequence, annotation files and protein sequences of the four species were downloaded from YamBase (https://yambase.org/organism/Dioscorea_alata/genome, accessed on 10 July 2023), Publikationen an der Universität Bielefeld (https://pub.uni-bielefeld.de/record/2941469#details, accessed on 28 February 2024), Iwate Biotechnology Research Center (https://genome-e.ibrc.or.jp/resource/yam, accessed on 28 February 2024) and CoGe (https://genomevolution.org/coge/GenomeInfo.pl?gid=54631, accessed on 28 February 2024), respectively.

### 4.2. Identification and Grouping of All RLKs

Proteins were acquired from primary transcripts across four selected species. In order to identify RLKs from genomes, HMMER v3.3.2 (http://hmmer.org/, accessed on 25 March 2024) was used in the identification of protein domains. First, the hmm model of the Pkinase domain (PF00069) was used to search all potential proteins with the kinase domain via hmmsearch (E-value cut-off: 1 × 10^−1^). Next, we scanned all domains presented in kinase proteins using the hmmscan (E-value cut-off: 1 × 10^−3^). Then, we predicted transmembrane domains via TMbed [49], which is a newly developed tool with more accuracy. One step was performed to reduce and rearrange all domains across a protein according to the descending layout of the E-value. All proteins that satisfied the specific structure were identified as RLKs.

### 4.3. Dividing RLKs into Different Subgroups

It has been reported that RLKs can be divided into different subgroups according to the phylogeny of RLK kinases [38]. We extracted kinase sequences from RLKs and blasted representative kinases of each subgroup using blastp (https://blast.ncbi.nlm.nih.gov/Blast.cgi, accessed on 25 March 2024). The RLK was classified into a specific subgroup considering the matched reference kinase belonging to [23]. RLKs among each subgroup were then filtered using the following criteria: (1) the kinase domain longer than 140 amino acids and (2) the kinase domain right after the transmembrane domain when multiple kinase domains existed.

### 4.4. Phylogeny Analysis of RLKs within and among Different Species

The multiple sequence alignment of RLKs within or across species was built by Mafft v7.45 [50]. The protein trees of *D. alata* RLKs and Dioscoreaceae RLKs were both built by IQ-TREE v1.6.12 [51] with the maximum likelihood method. Phylogenetic tree visualization was conducted by iTOLs Version 6.9 (https://itol.embl.de/, accessed on 26 March 2024) [52].

### 4.5. Gene Chromosome Location and Cluster Analysis

All information on the RLK gene location was obtained from the *D. alata* genome annotation GFF file. The genetic mapping of RLKs located on chromosomes was generated via TBtools-II v2.078 [53]. The gene cluster was defined from the following criteria: if two genes were located within 200 kb, then they would be treated in order to be located within a cluster. Gene clusters were also mapped to different chromosomes. The plot was generated by a Perl5 script.

### 4.6. Duplication Events and Synteny Analysis

The protein file of *D. alata* was searched by MCScanX [54] utilizing blastp, with the help of the GFF annotation and synteny analysis within the *D. alata*. Circle plot and wiring between different related genes were completed using the TBtools-II [53]. Gene pairs that belonged to the segmental duplication event were also calculated for their Ks and Ka/Ks ratio via TBtools-II [53].

### 4.7. Ancestral Gene Reconstruction

The ancestral gene reconstruction was conducted by reconciling the gene tree and the species tree of four species from the Dioscoreaceae. Gene loss/duplication events of RLK genes, which existed among four species, were inferred by Notung-2.9.1.5 [55].

### 4.8. RNA Extraction and Gene Expression Analysis

The leaves of *D. alata* inoculated with *C. gloeosporioides* for 24 and 48 h were treated as the experiment group, whereas those uninoculated were used as the control. Total RNA was extracted using Trizol (10296010, Invitrogen, Carlsbad, USA) according to the manufacturer’s suggestion. RNA-seq was conducted in triplicate, with each sample made up of three individual plants at Wuhan Benagen Tech Solutions Company Limited, Wuhan, China. After obtaining the raw sequencing data, the adapters in RNA-seq data were removed by Trim galore v0.6.4 (https://github.com/FelixKrueger/TrimGalore, accessed on 10 July 2023). Clean reads were mapped to the reference genome of *D. alata* by Hisat2 v2.1.0 [56]. Finally, gene expression matrices were quantified using FeatureCounts v2.0.1 [57], from which all genes were calculated and normalized to FPKM.

### 4.9. Searching for Differentially Expressed Genes

Differentially expressed genes (DEGs) among *D.alata* RLKs were acquired using the R package DEseq2 [58]. In order to find genes induced or reduced by *C. gloeosporioides*, we analyzed DEGs of RLK by comparing 24 h post-inoculation (hpi) and 48 hpi in the control group, respectively. The FPKM was presented as the heatmap around the RLK phylogeny tree of *D. alata* using iTOL [52]. The volcano plot of DEGs and the bar plot of DEGs subgroup were both painted via GraphPad Prism 8.0 (https://www.graphpad.com/, accessed on 28 March 2024).

## Data Availability

The clean reads of the RNA-seq were deposited into the National Genomics Data Center (PRJCA025737). All other relevant data are contained within the article.

## Supplementary Materials

The following supporting information can be downloaded at: https://www.mdpi.com/article/10.3390/plants13091274/s1. Figure S1: The number of total RLK genes and their subgroups among four species of Dioscoreaceae; Figure S2: The phylogeny of RLK genes in *D. alata*; Figure S3: Physical distribution of RLKs on chromosomes of *D. alata*; Table S1: Detailed subgroup information of RLK genes identified from *D. alata*; Table S2: The number of RLKs and their subgroups of four species of Dioscoreaceae; Table S3: The Ks value and Ka/Ks ratio of each RLK pair generated by WGDs or SDs; Table S4: Stress-related cis-acting elements of each RLK gene promoter region of *D. alata*; Table S5: Statistics of the stress-related cis-acting elements of RLK gene subgroups in the *D. alata*; Table S6: Differentially expressed RLK genes in *D. alata* inoculated by *C. gloeosporioides* at 24 hpi; Table S7: Differentially expressed RLK genes in *D. alata* inoculated by *C. gloeosporioides* at 48 hpi.

## References

[1] Sharif B.M., Burgarella C., Cormier F., Mournet P., Causse S., Van K.N., Kaoh J., Rajaonah M.T., Lakshan S.R., Waki J. (2020). Genome-wide genotyping elucidates the geographical diversification and dispersal of the polyploid and clonally propagated yam (*Dioscorea alata*). Ann. Bot..

[2] Cormier F., Martin G., Vignes H., Lachman L., Cornet D., Faure Y., Maledon E., Mournet P., Arnau G., Chaïr H. (2021). Genetic control of flowering in greater yam (*Dioscorea alata* L.). BMC Plant Biol..

[3] Lebot V., Lawac F., Legendre L. (2023). The greater yam (*Dioscorea alata* L.): A review of its phytochemical content and potential for processed products and biofortification. J. Food Compos. Anal..

[4] Wang P.T., Shan N., Ali A., Sun J.Y., Luo S., Xiao Y., Wang S.L., Hu R., Huang Y.J., Zhou Q.H. (2022). Comprehensive evaluation of functional components, biological activities, and minerals of yam species (*Dioscorea polystachya* and *D. alata*) from China. LWT.

[5] Wang Y., Lu R.S., Li M.H., Lu X.Y., Sun X.Q., Zhang Y.M. (2024). Unraveling the Molecular Basis of Color Variation in *Dioscorea alata* Tubers: Integrated Transcriptome and Metabolomics Analysis. Int. J. Mol. Sci..

[6] Salehi B., Sener B., Kilic M., Sharifi-Rad J., Naz R., Yousaf Z., Mudau F.N., Fokou P.V.T., Ezzat S.M., El Bishbishy M.H. (2019). *Dioscorea* Plants: A Genus Rich in Vital Nutra-pharmaceuticals-A Review. Iran. J. Pharm. Res..

[7] Maithili V., Dhanabal S.P., Mahendran S., Vadivelan R. (2011). Antidiabetic activity of ethanolic extract of tubers of *Dioscorea alata* in alloxan induced diabetic rats. Indian. J. Pharmacol..

[8] Mahmad N., Taha R.M., Othman R., Abdullah S., Anuar N., Elias H., Rawi N. (2018). Anthocyanin as potential source for antimicrobial activity in *Clitoria ternatea* L. and *Dioscorea alata* L.. Pigm Resin. Technol..

[9] Egesi C.N., Onyeka T.J., Asiedu R. (2007). Severity of anthracnose and virus diseases of water yam (*Dioscorea alata* L.) in Nigeria I: Effects of yam genotype and date of planting. Crop Prot..

[10] Korada R.R., Naskar S.K., Edison S. (2010). Insect pests and their management in yam production and storage: A world review. Int. J. Pest. Manag..

[11] Penet L., Cornet D., Blazy J.M., Alleyne A., Barthe E., Bussière F., Guyader S., Pavis C., Pétro D. (2016). Varietal Dynamics and Yam Agro-Diversity Demonstrate Complex Trajectories Intersecting Farmers’ Strategies, Networks, and Disease Experience. Front. Plant Sci..

[12] Wang Y., Xu W.T., Lu R.S., Chen M., Liu J., Sun X.Q., Zhang Y.M. (2023). Genome sequence resource for *Colletotrichum gloeosporioides*, an important pathogenic fungus causing anthracnose of *Dioscorea alata*. Plant Dis..

[13] Ntui V.O., Uyoh E.A., Ita E.E., Markson A.A.A., Tripathi J.N., Okon N.I., Akpan M.O., Phillip J.O., Brisibe E.A., Ene-Obong E.O.E. (2021). Strategies to combat the problem of yam anthracnose disease: Status and prospects. Mol. Plant Pathol..

[14] Abraham K., Nemorin A., Lebot V., Arnau G. (2013). Meiosis and sexual fertility of autotetraploid clones of greater yam *Dioscorea alata* L.. Genet. Resour. Crop Evol..

[15] Tor M., Ainsworth C., Mantell S.H. (1993). Stable Transformation of the Food Yam *Dioscorea alata* L. by Particle Bombardment. Plant Cell Rep..

[16] Syombua E.D., Zhang Z.Z., Tripathi J.N., Ntui V.O., Kang M., George O.O., Edward N.K., Wang K., Yang B., Tripathi L. (2021). A CRISPR/Cas9-based genome-editing system for yam (*Dioscorea* spp.). Plant Biotechnol. J..

[17] Ngou B.P.M., Ding P.T., Jones J.D.G. (2022). Thirty years of resistance: Zig-zag through the plant immune system. Plant Cell.

[18] Kourelis J., van der Hoorn R.A.L. (2018). Defended to the Nines: 25 Years of Resistance Gene Cloning Identifies Nine Mechanisms for R Protein Function. Plant Cell.

[19] Boutrot F., Zipfel C. (2017). Function, Discovery, and Exploitation of Plant Pattern Recognition Receptors for Broad-Spectrum Disease Resistance. Annu. Rev. Phytopathol..

[20] Sun Y.C., Wang X.J., Liu F.Y., Guo H.Y., Wang J.F., Wei Z.T., Kang Z.S., Tang C.L. (2023). A Leucine-Rich Repeat Receptor-like Kinase TaBIR1 Contributes to Wheat Resistance against *Puccinia striiformis* f. sp. *tritici*. Int. J. Mol. Sci..

[21] Dambroz C.M.D., Aono A.H., Silva E.M.D., Pereira W.A. (2023). Genome-wide analysis and characterization of the LRR-RLK gene family provides insights into anthracnose resistance in common bean. Sci. Rep..

[22] Bisneta M.V., Gonçalves-Vidigal M.C. (2020). Integration of anthracnose resistance loci and RLK and NBS-LRR-encoding genes in the *Phaseolus vulgaris* L. genome. Crop Sci..

[23] Lehti-Shiu M.D., Shiu S.H. (2012). Diversity, classification and function of the plant protein kinase superfamily. Philos. Trans. R. Soc. B Biol. Sci..

[24] Meyers B.C., Kozik A., Griego A., Kuang H.H., Michelmore R.W. (2003). Genome-wide analysis of NBS-LRR-encoding genes in Arabidopsis. Plant Cell.

[25] Shao Z.Q., Xue J.Y., Wu P., Zhang Y.M., Wu Y., Hang Y.Y., Wang B., Chen J.Q. (2016). Large-Scale Analyses of Angiosperm Nucleotide-Binding Site-Leucine-Rich Repeat Genes Reveal Three Anciently Diverged Classes with Distinct Evolutionary Patterns. Plant Physiol..

[26] Qiao X., Li Q.H., Yin H., Qi K.J., Li L.T., Wang R.Z., Zhang S.L., Paterson A.H. (2019). Gene duplication and evolution in recurring polyploidization-diploidization cycles in plants. Genome Biol..

[27] Panchy N., Lehti-Shiu M., Shiu S.H. (2016). Evolution of Gene Duplication in Plants. Plant Physiol..

[28] Cui L., Wall P.K., Leebens-Mack J.H., Lindsay B.G., Soltis D.E., Doyle J.J., Soltis P.S., Carlson J.E., Arumuganathan K., Barakat A. (2006). Widespread genome duplications throughout the history of flowering plants. Genome Res..

[29] Vanneste K., Baele G., Maere S., Van de Peer Y. (2014). Analysis of 41 plant genomes supports a wave of successful genome duplications in association with the Cretaceous–Paleogene boundary. Genome Res..

[30] Soltabayeva A., Dauletova N., Serik S., Sandybek M., Omondi J.O., Kurmanbayeva A., Srivastava S. (2022). Receptor-like Kinases (LRR-RLKs) in Response of Plants to Biotic and Abiotic Stresses. Plants.

[31] Ngou B.P.M., Heal R., Wyler M., Schmid M.W., Jones J.D.G. (2022). Concerted expansion and contraction of immune receptor gene repertoires in plant genomes. Nat. Plants.

[32] Yu H., Bai F., Ji C., Fan Z., Luo J., Ouyang B., Deng X., Xiao S., Bisseling T., Limpens E. (2023). Plant lysin motif extracellular proteins are required for arbuscular mycorrhizal symbiosis. Proc. Natl. Acad. Sci. USA.

[33] Ivanov S., Harrison M.J. (2024). Receptor-associated kinases control the lipid provisioning program in plant-fungal symbiosis. Science.

[34] Thapa G., Gunupuru L.R., Hehir J.G., Kahla A., Mullins E., Doohan F.M. (2018). A Pathogen-Responsive Leucine Rich Receptor Like Kinase Contributes to *Fusarium* Resistance in Cereals. Front. Plant Sci..

[35] Wang J., Hao F.S., Song K.F., Jin W.H., Fu B., Wei Y.F., Shi Y.C., Guo H.X., Liu W.Q. (2020). Identification of a Novel *NtLRR*-*RLK* and Biological Pathways That Contribute to Tolerance of TMV in *Nicotiana tabacum*. Mol. Plant Microbe Interact..

[36] Han P.L., Li R., Yue Q.Y., Li F.D., Nie J.J., Yin Z.Y., Xu M., Guan Q.M., Huang L.L. (2022). The Apple Receptor-Like Kinase MdSRLK3 Positively Regulates Resistance against Pathogenic Fungus *Valsa mali* by Affecting the Ca^2+^ Signaling Pathway. Phytopathology.

[37] Ngou B.P.M., Wyler M., Schmid M.W., Kadota Y., Shirasu K. (2024). Evolutionary trajectory of pattern recognition receptors in plants. Nat. Commun..

[38] Shiu S.H., Bleecker A.B. (2001). Receptor-like kinases from *Arabidopsis* form a monophyletic gene family related to animal receptor kinases. Proc. Natl. Acad. Sci. USA.

[39] Lehti-Shiu M.D., Zou C., Hanada K., Shiu S.H. (2009). Evolutionary History and Stress Regulation of Plant Receptor-Like Kinase/Pelle Genes. Plant Physiol..

[40] Shao Z.Q., Zhang Y.M., Hang Y.Y., Xue J.Y., Zhou G.C., Wu P., Wu X.Y., Wu X.Z., Wang Q., Wang B. (2014). Long-Term Evolution of Nucleotide-Binding Site-Leucine-Rich Repeat Genes: Understanding Gained from and beyond the Legume Family. Plant Physiol..

[41] Li X.T., Feng X.Y., Zeng Z., Liu Y., Shao Z.Q. (2021). Comparative Analysis of HSF Genes from Secale cereale and its Triticeae Relatives Reveal Ancient and Recent Gene Expansions. Front. Genet..

[42] Zhou F.L., Guo Y., Qiu L.J. (2016). Genome-wide identification and evolutionary analysis of leucine-rich repeat receptor-like protein kinase genes in soybean. BMC Plant Biol..

[43] Wang J.L., Hu T.H., Wang W.H., Hu H.J., Wei Q.Z., Bao C.L. (2019). Investigation of evolutionary and expressional relationships in the function of the leucine-rich repeat receptor-like protein kinase gene family (LRR-RLK) in the radish (*Raphanus sativus* L.). Sci. Rep..

[44] Meng J., Yang J., Peng M.D., Liu X.L., He H.B. (2020). Genome-Wide Characterization, Evolution, and Expression Analysis of the Leucine-Rich Repeat Receptor-Like Protein Kinase (LRR-RLK) Gene Family in *Medicago truncatula*. Life.

[45] Bredeson J.V., Lyons J.B., Oniyinde I.O., Okereke N.R., Kolade O., Nnabue I., Nwadili C.O., Hribova E., Parker M., Nwogha J. (2022). Chromosome evolution and the genetic basis of agronomically important traits in greater yam. Nat. Commun..

[46] Siadjeu C., Pucker B., Viehoever P., Albach D.C., Weisshaar B. (2020). High Contiguity de novo Genome Sequence Assembly of Trifoliate Yam (*Dioscorea dumetorum*) Using Long Read Sequencing. Genes.

[47] Tamiru M., Natsume S., Takagi H., White B., Yaegashi H., Shimizu M., Yoshida K., Uemura A., Oikawa K., Abe A. (2017). Genome sequencing of the staple food crop white Guinea yam enables the development of a molecular marker for sex determination. BMC Biol..

[48] Chellappan B.V., Shidhi P.R., Vijayan S., Rajan V.S., Sasi A., Nair A.S. (2019). High Quality Draft Genome of Arogyapacha (*Trichopus zeylanicus*), an Important Medicinal Plant Endemic to Western Ghats of India. G3-Genes Genomes Genet..

[49] Bernhofer M., Rost B. (2022). TMbed: Transmembrane proteins predicted through language model embeddings. BMC Bioinform..

[50] Katoh K., Standley D.M. (2013). MAFFT Multiple Sequence Alignment Software Version 7: Improvements in Performance and Usability. Mol. Biol. Evol..

[51] Nguyen L.T., Schmidt H.A., Von Haeseler A., Minh B.Q. (2015). IQ-TREE: A Fast and Effective Stochastic Algorithm for Estimating Maximum-Likelihood Phylogenies. Mol. Biol. Evol..

[52] Letunic I., Bork P. (2021). Interactive Tree Of Life (iTOL) v5: An online tool for phylogenetic tree display and annotation. Nucleic Acids Res..

[53] Chen C., Wu Y., Li J., Wang X., Zeng Z., Xu J., Liu Y., Feng J., Chen H., He Y. (2023). TBtools-II: A “one for all, all for one” bioinformatics platform for biological big-data mining. Mol. Plant.

[54] Wang Y., Tang H., De Barry J.D., Tan X., Li J., Wang X., Lee T.-H., Jin H., Marler B., Guo H. (2012). *MCScanX*: A toolkit for detection and evolutionary analysis of gene synteny and collinearity. Nucleic Acids Res..

[55] Chen K., Durand D., Farach-Colton M. (2000). NOTUNG: A program for dating gene duplications and optimizing gene family trees. J. Comput. Biol..

[56] Kim D., Paggi J.M., Park C., Bennett C., Salzberg S.L. (2019). Graph-based genome alignment and genotyping with HISAT2 and HISAT-genotype. Nat. Biotechnol..

[57] Pertea G., Pertea M. (2020). GFF Utilities: GffRead and GffCompare. F1000Research.

[58] Love M.I., Huber W., Anders S. (2014). Moderated estimation of fold change and dispersion for RNA-seq data with DESeq2. Genome Biol..

