# Supplementary material for: Genome-Wide Identification and Evolutionary Analysis of Receptor-like Kinase Family Genes Provides Insights into Anthracnose Resistance of Dioscorea alata
Source: Plants (Basel). 2024 May 5;13(9):1274. doi: 10.3390/plants13091274 (PMC11085297; doi:10.3390/plants13091274)
Supplement: Supplementary file 1 [file plants-13-01274-s001.zip › Figure S2.pdf]

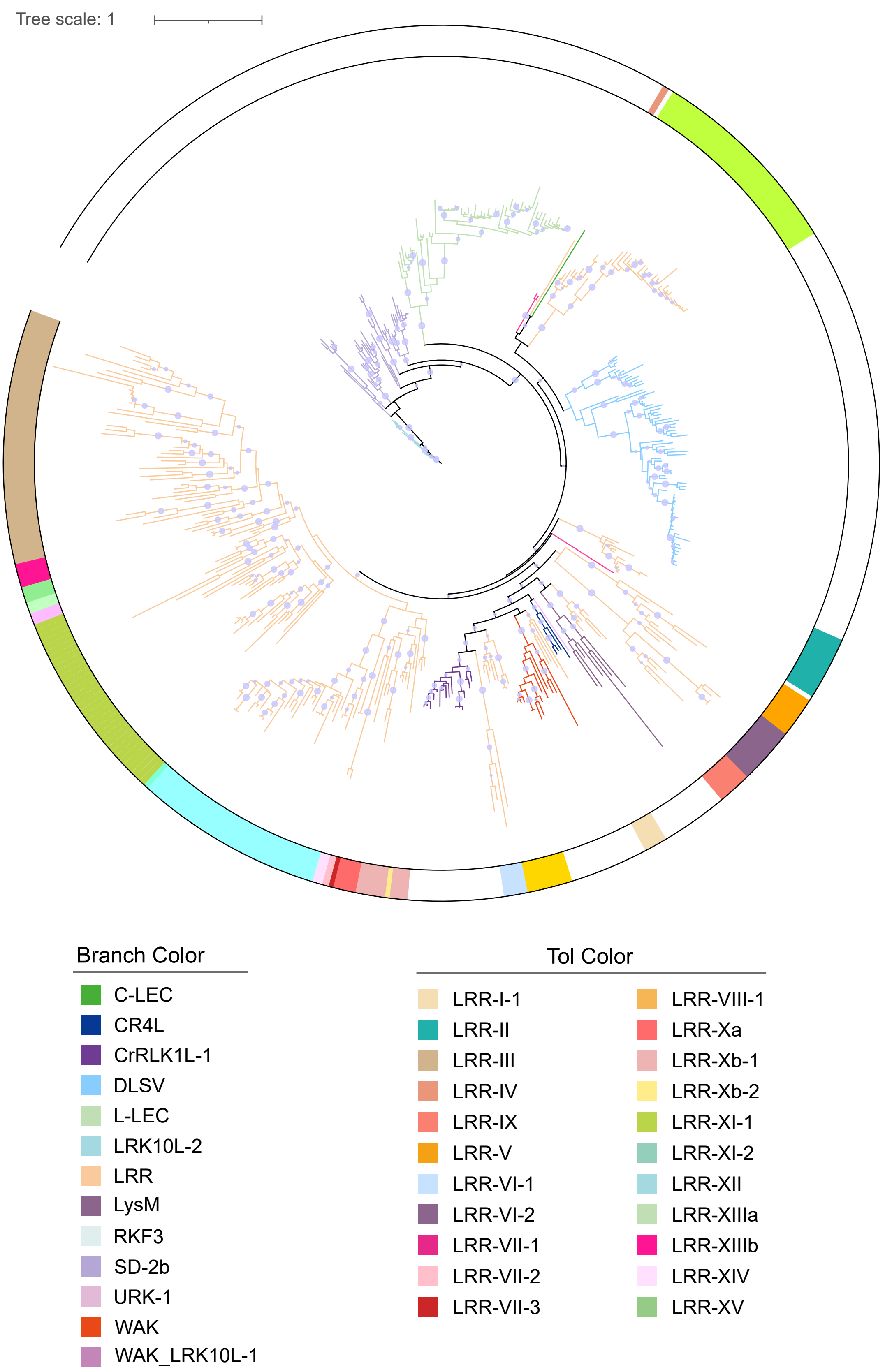

Tree scale: 1
Branch Color
C-LEC
CR4L
CrRLK1L-1
DLSV
L-LEC
LRK10L-2
LRR
LysM
RKF3
SD-2b
URK-1
WAK
WAK_LRK10L-1
Tol Color
LRR-I-1
LRR-II
LRR-III
LRR-IV
LRR-IX
LRR-V
LRR-VI-1
LRR-VI-2
LRR-VII-1
LRR-VII-2
LRR-VII-3
LRR-VIII-1
LRR-Xa
LRR-Xb-1
LRR-Xb-2
LRR-XI-1
LRR-XI-2
LRR-XII
LRR-XIIIa
LRR-XIIIb
LRR-XIV
LRR-XV
