# Supplementary figures and images for: Genome-Wide Identification and Evolutionary Analysis of Receptor-like Kinase Family Genes Provides Insights into Anthracnose Resistance of Dioscorea alata
Source: Plants (Basel). 2024 May 5;13(9):1274. doi: 10.3390/plants13091274 (PMC11085297; doi:10.3390/plants13091274)

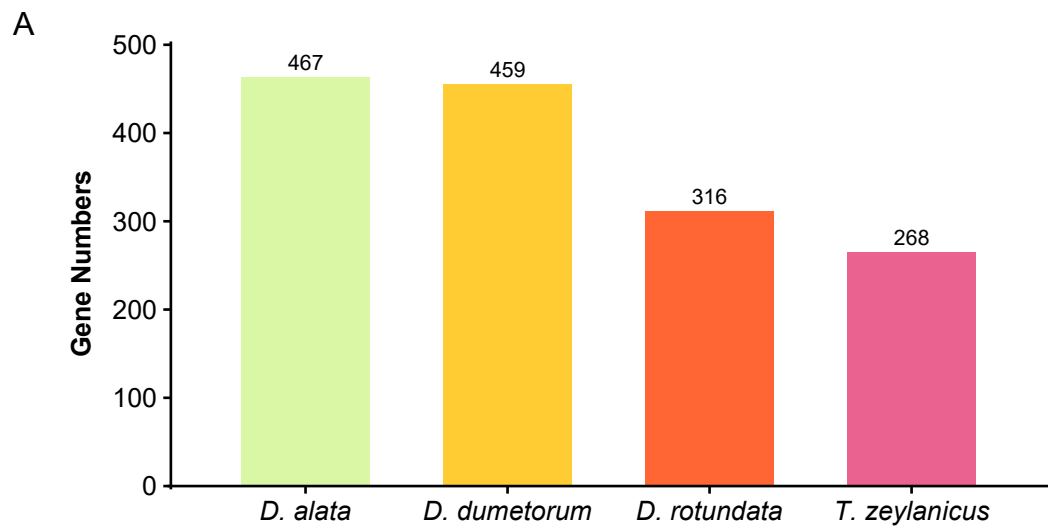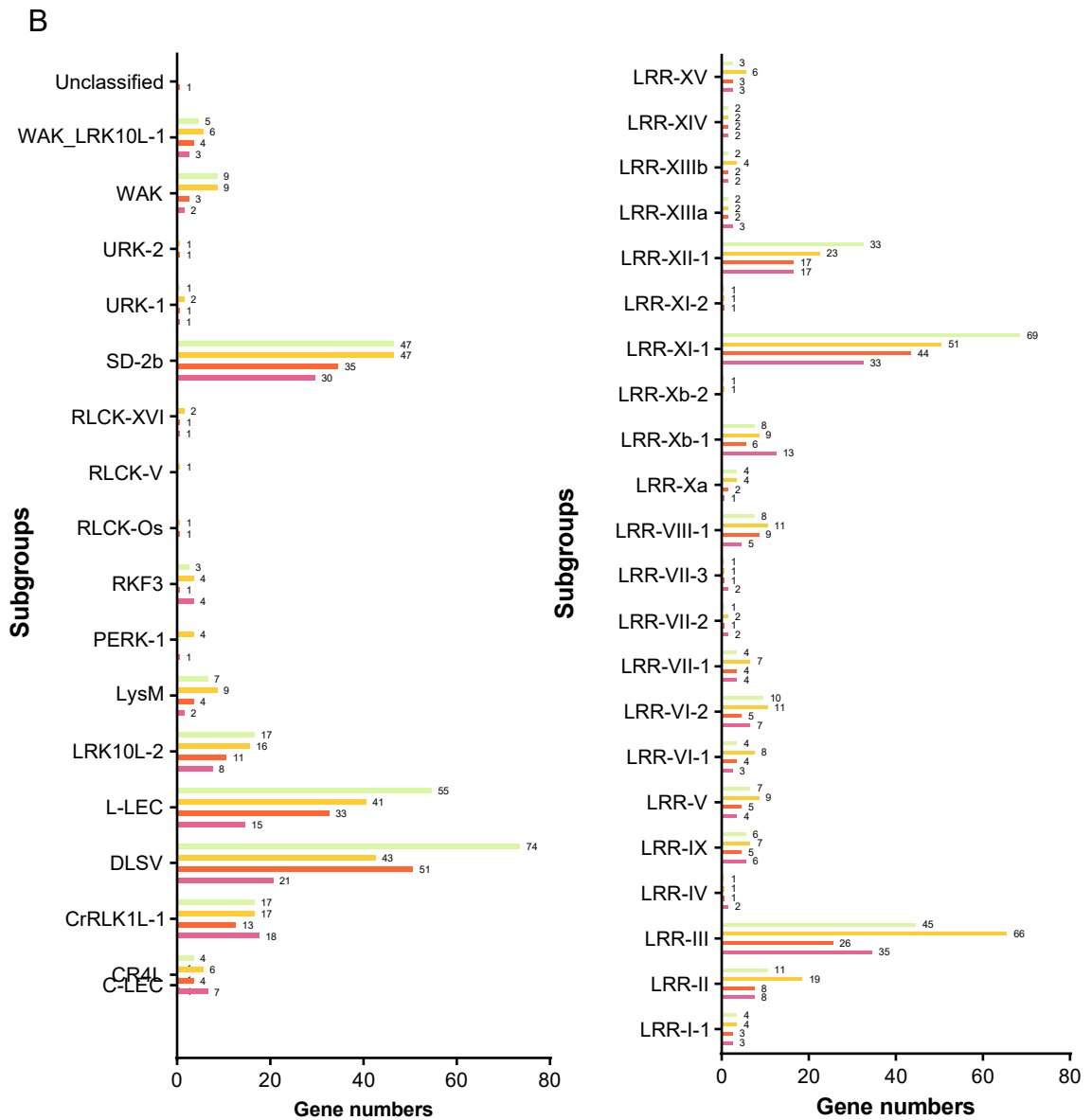

Supplement: Supplementary file 1 [file plants-13-01274-s001.zip › Figure S1.pdf]

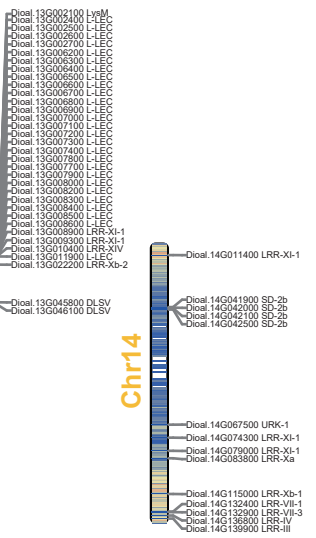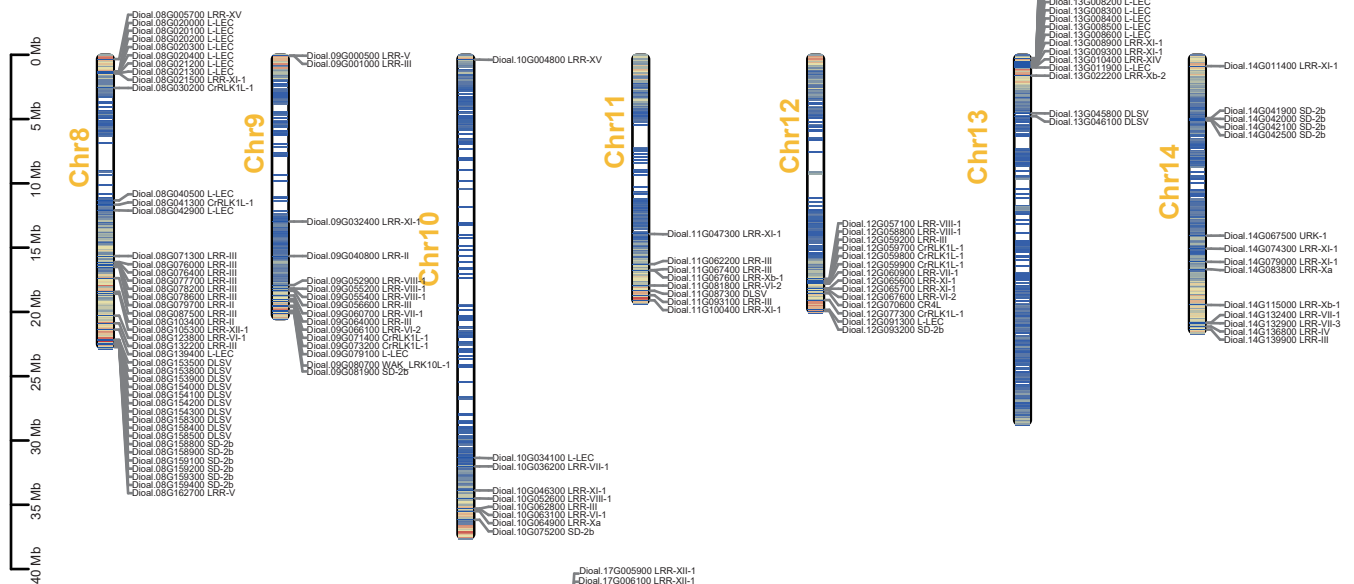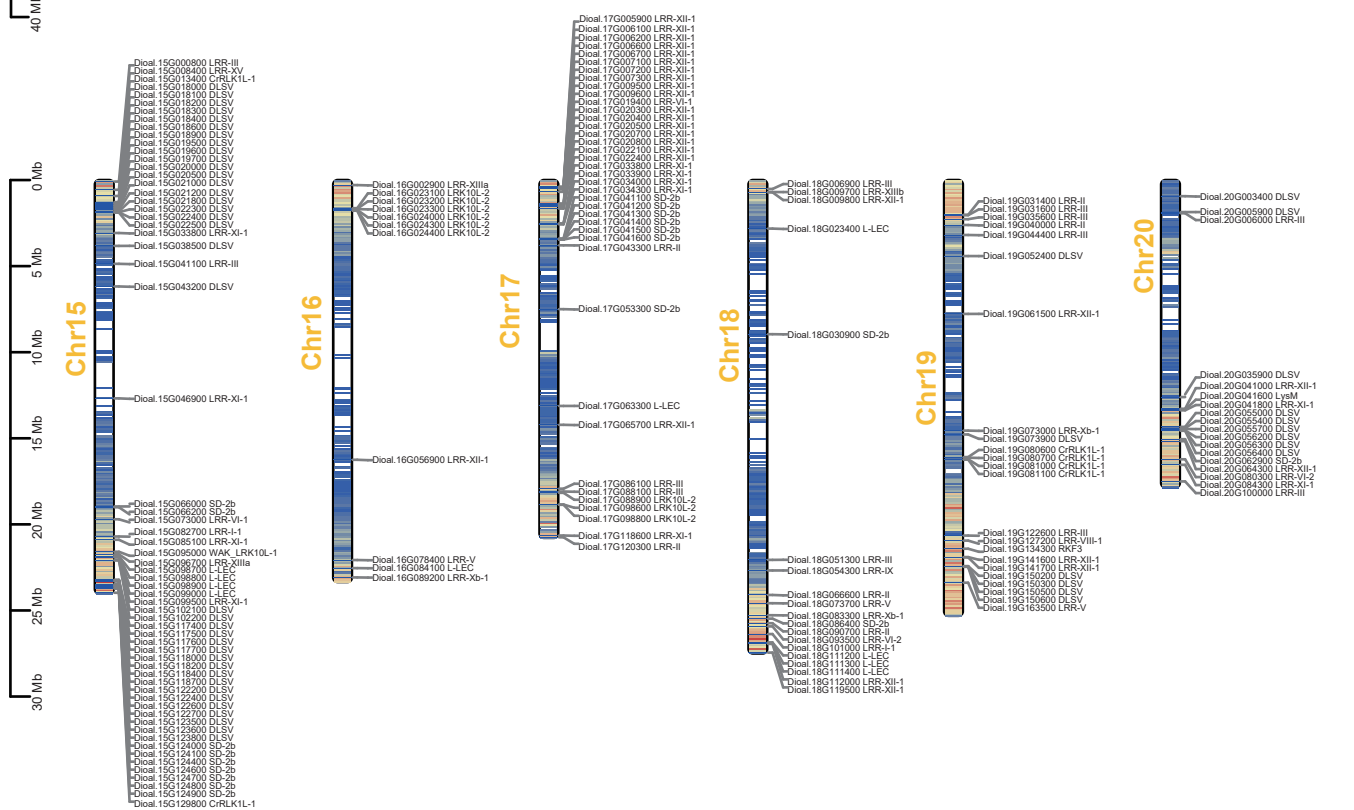

Supplement: Supplementary file 1 [file plants-13-01274-s001.zip › Figure S3.pdf]
